# Can Retrospective Reports Provide Accurate Job History Information? A Comparison With Concurrent Reports in a National Prospective Study of Older Adults

**DOI:** 10.1093/geroni/igae021

**Published:** 2024-02-23

**Authors:** Amanda Sonnega, Maymona Al-Hinai, Qize Chen, Brooke Helppie-McFall, Jacqui Smith

**Affiliations:** Institute for Social Research, University of Michigan, Ann Arbor, Michigan, USA; School of Public Health, University of Michigan, Ann Arbor, Michigan, USA; Department of Economics, University of Michigan, Ann Arbor, Michigan, USA; Institute for Social Research, University of Michigan, Ann Arbor, Michigan, USA; Institute for Social Research, University of Michigan, Ann Arbor, Michigan, USA

**Keywords:** Employment history, Prospective, Validation

## Abstract

**Background and Objectives:**

The growing interest in the impact of lifetime occupational exposures on later-life health underscores the need to expand and evaluate the quality of data resources. The present study took advantage of a retrospective life history survey fielded within the context of the Health and Retirement Study to assess the accuracy of retrospectively obtained information on job history. We evaluated hypotheses related to job history and respondent characteristics to understand more about factors associated with recall accuracy.

**Research Design and Methods:**

We used data from the Life History Mail Survey (LHMS), a self-administered survey conducted in 2015 and 2017. We compared the match rate of work status collected in the LHMS questionnaire with data collected concurrently during HRS core face-to-face or phone interviews from 1992 through 2016 with respect to jobs held at the time of the interview. We also conducted a limited set of comparisons of occupation and industry match.

**Results:**

The sample was 61.79% women, 82.12% White, and 8.57% Hispanic with a mean age of 74.70 years. The overall work status match rate was 83%. Jobs held longer ago were recalled with less accuracy. Jobs held for longer durations and that were full-time rather than part-time were recalled with greater accuracy. More complex job histories that involved a larger number of jobs were also associated with a lower match rate. Higher levels of conscientiousness and cognitive functioning were both associated with a higher match between the two sources of work status information. The occupation match rate was 69%, and the industry match rate was 77%.

**Discussion and Implications:**

A self-administered, paper-and-pencil questionnaire attempting to measure decades-long histories of autobiographically important dimensions of life can provide reasonably accurate historical employment information. Several factors are likely to influence the relative accuracy of recalled information.

**Translational Significance:** Growing interest in the impact of lifetime occupational exposures on later life health and well-being has underscored the need to expand and evaluate the quality of data resources for research. A self-administered, paper-and-pencil questionnaire attempting to measure decades-long histories of autobiographically important dimensions of life can provide reasonably accurate historical employment information. This information can be used to evaluate the effects of a wide range of occupational exposures on important later life outcomes such as functional capacity and cognitive functioning.

The influence of life course exposures on later life outcomes is gaining increased research attention. A growing literature examines occupational experiences and later life outcomes, for example, cognitive functioning (Fujishiro et al., 2019) and disability (Nicholas et al., 2020). These studies have been limited in their ability to characterize the entire life course, however, especially with regard to lifetime occupational exposures. Further research progress will benefit from the growing availability of complete life histories. This information can be obtained from individuals either concurrently or retrospectively, each method having a set of well-known benefits and liabilities for epidemiologic research (Sonnega & Sonnega, 2023). Retrospective surveys can obtain information efficiently but are more subject to recall bias, whereas information obtained concurrently/prospectively will be less subject to recall bias but can take years to collect and be quite expensive. The present study takes advantage of a retrospective life history survey that took place within a prospective survey, the Health and Retirement Study (HRS), to evaluate the accuracy of retrospectively obtained information on lifetime job history.

The HRS is a national longitudinal biennial survey of adults in the United States that has been ongoing since 1992. The data offer a wealth of information collected concurrently about many aspects of the lives of participants from their entry into the study over age 50 until their death or study attrition. To fill the gaps in earlier life history, the HRS created and fielded the Life History Mail Survey (LHMS), a self-administered, paper-and-pencil questionnaire that collected information about respondents’ life histories up to age 50. Researchers have begun to validate the information obtained in the LHMS (Smith et al., 2021) but none to date have reported on the accuracy of the job history reports.

In this study, we examine the accuracy of factual information obtained retrospectively in the LHMS by comparing job histories collected in the LHMS questionnaire with data collected concurrently during HRS face-to-face or phone interviews from 1992 through 2016 with respect to jobs held at the time of interview. Concurrent reports are less subject to recall bias and so are likely more representative of the ground truth. We evaluate how well information from the LHMS on work status matches with the concurrent reports and how the match rates differ along dimensions suggested by theories of autobiographical memory.

Finally, while other panel studies have included retrospective assessment of life history information (Havari & Mazzonna, 2015), to our knowledge, the LHMS represents the first time a self-administered paper-and-pencil assessment of detailed life history information has been implemented. This is, therefore, the first time retrospective job history information obtained in this manner has been validated against concurrent reports collected prospectively by interview.

## Autobiographical Memory and the Challenge of Recall

As Schwarz and Sudman (1994) noted in their introduction to *Autobiographical Memory and the Validity of Retrospective Reports*, cognitive psychologists began developing theories of autobiographical memory relatively recently, beginning in the 1970s. At the same time, scholars of survey methods research focused on seeking answers to how and why people provide information that is, by turns, accurate or inaccurate in their survey responses. The confluence of these separate lines of inquiry led to significant advances in our understanding, particularly, of retrospective behavioral reports. A high-level summary of this literature suggests that individuals, in general, are reliable reporters of events in their personal history, but a wide range of factors are likely to influence the relative accuracy of recalled information. Some of these factors are amenable to influence by survey researchers attempting to elicit retrospective reports. Others are not subject to manipulation but can be accounted for analytically (if known).

We relied on theory and research in autobiographical memory during the instrument development phase of the LHMS to inform potential methods to improve recall. For example, literature finds that events are often linked in memory to form “autobiographical sequences” that preserve relative order of information (Bradburn et al., 1987). This suggests that providing a structure that prompts the relative order of events is likely to promote better recall. Thus, the LHMS provided a job history grid that elicited up to 10 jobs, beginning with the first job held for at least 1 year after leaving full-time schooling and proceeding chronologically. We asked the “easiest” to remember information first—what the job was—with the more difficult information—the dates the job was held—coming second. To further assist the reconstruction of the autobiographical sequence, the third set of questions for each job asked what happened after leaving that job (i.e., started another job, took medical/disability leave, unemployed, student, etc.).

Theories of autobiographical memory also guided our hypothesis testing. Autobiographical memory theory proposes a developmental framework whereby individuals are born into, grow up, and age in social and cultural contexts, and steadily, over their life course, develop a personal narrative of their own experiences and lives (Nelson & Fivush, 2020). Some aspects of autobiographical memory theory can directly inform our thinking on how individuals are likely to perform on surveys that challenge their memory. Strength theories assume that each memory has a unique strength that declines over time (Friedman, 1993). Over time, one’s memory of any given event becomes weaker or less accurate. This recall decay suggests there may be an inability to recall events that occured in the distant past (Ricker et al., 2020). This implies that in longitudinal panel studies the accuracy of reports of an event should decrease as time increases since the event. That is, jobs held longer ago will be more difficult to recall accurately. Similarly, the feature of salience in autobiographical memory suggests that events and experiences that were more important for individuals are likely to be remembered with greater accuracy (Rubin & Kozin, 1984). For example, jobs held for longer periods or that were full-time may be more easily recalled. Last, recounting a full lifetime job history is likely to be a difficult task, especially for more complex job histories involving multiple jobs.

Research shows that memory about when an event took place is often inexact (Belli, 1998); however, when the recall task demands more precision than memory itself can provide, respondents use a variety of processes to supplement what is stored in memory with whatever can be reconstructed or inferred at the time the question is asked (Belli, 1998). Event history calendars leverage this facility by providing multiple life contexts that can act synergistically to cue memory and aid recall. For example, asking about residential history at the same time as occupational history might improve recall of the exact dates of both if, for example, a move occurred in relation to a job change. A major advantage of the self-administered nature of the LHMS (with no time constraint), is that respondents were free to consult their own records to improve the accuracy of their reports if they were able and motivated to do so. Thus, personal characteristics such as level of cognitive functioning and conscientiousness likely come into play in completing the LHMS job history section.

This background led us to propose the following testable hypotheses regarding features of job history and personal characteristics in relation to the match between reports of work status (working or not) in the LHMS compared to the HRS core with respect to jobs held at the time of the HRS core interviews.

## Job History Hypotheses

*H1a*: We will observe lower match rates for job histories that include a larger number of jobs.

*H1b*: We will observe lower match rates for jobs that were held further back in time from the date of LHMS data collection (2017).

*H1c*: We will observe higher match rates for jobs that were held full-time.

*H1d*: We will observe higher match rates for jobs held for longer periods of time.

## Personal Characteristics Hypotheses

*H2a*: We will observe a positive association between match rates and cognitive functioning at the time of the LHMS.

*H2b*: We will observe with a positive association between match rates and respondents’ self-reported conscientiousness at the time of the LHMS.

## Method

### Sample

The HRS is a national longitudinal biennial study of adults over age 50 in the United States. The study began in 1992 with the original HRS cohort (born 1931–1941) then aged 51–61. Cohorts were added subsequently, and in 1998, the HRS instituted a steady-state design adding a new cohort every 6 years. The survey includes rich measurements of factors affecting well-being across the retirement transition and onward. Details are provided in Sonnega et al. (2014). The currently enrolled cohorts include the Assets and Health Dynamics of the Oldest Old (AHEAD), born earlier than 1924 and enrolled in 1993; the HRS, born 1931–1941 and enrolled in 1992; the Children of the Depression (CODA), born 1926–1930, and the War Babies, born 1942–1947, both enrolled in 1998; the Early Baby Boomers, born 1948–1953 and enrolled in 2004, the Mid Baby Boomers, born 1954–1959 and enrolled in 2010, the Late Baby Boomers, born 1960–1965 and enrolled in 2016, and Early GenX, born 1966–1971 and enrolled in 2022. The initial fielding of the LHMS in 2015 and in 2017 included the AHEAD through Mid Baby Boomer cohorts. To reduce respondent burden, the Late Baby Boomers were sent the LHMS in 2019. All new cohorts will be administered this one-time life-history assessment close to their enrollment wave. We used the available LHMS data from the cross-wave 2015–2017 LHMS Harmonized and Aggregated Public Data (Larkina et al., 2021; Smith et al., 2023a) in conjunction with employment information from the core for 1992 through 2016 (*n* = 11.775).

We drew information on sociodemographic and personal characteristics as well as concurrent employment histories from the RAND HRS Longitudinal File, a cleaned and processed version of much of the HRS core biennial interview data (Bugliari et al., 2023). We used imputed cognition data from the Langa–Weir data that provide a summary score for cognition for each biennial wave from 1992 to 2020 (Langa et al., 2023). We drew one variable (conscientiousness) from the psychosocial leave-behind questionnaire (Smith et al., 2023b). The HRS is sponsored by the National Institute on Aging (grant number NIA U01AG009740) and is conducted by the University of Michigan (UM). All protocols have been approved by the UM Institutional Review Board.

The total sample size of the combined data set was 10,468 individuals, representing 87,048 person-years of observation. We removed 180 overlap cases (between the AHEAD and HRS cohorts) and 449 cases in the Late Baby Boomer cohort due to the very small sample sizes of this cohort in the LHMS. We further restricted to cases that had observations for all variables in multivariate analyses yielding a final analytic sample of 8,648 individuals with 74,751 person-years of observation. See Table 1 for the descriptive statistics for all study variables in the total sample and by match status.

**Table 1.** Descriptive Statistics for all Study Variables in the Total Sample and by Match Status (*N* = 74,751 Person-Years Representing 8,648 Individuals)

Study variables	Total sample	Not matching	Matching	Cohen’s D
Respondent characteristics				
Age (mean, *SE*)	74.70 (8.70)	74.80 (8.30)	74.60 (8.80)	−0.013
Gender (%)				
Male	38.21	18.86	81.14	(base)
Female	61.79	16.92	83.08	0.049
Race (%)				
White/Caucasian	82.12	17.07	82.93	(base)
Black/African American	13.20	20.83	79.17	−0.100
Other	4.68	19.07	80.93	−0.053
Ethnicity (%)				
Non-Hispanic	91.43	17.34	82.66	(base)
Hispanic	8.57	21.17	78.83	−0.101
Cohort (%)				
AHEAD	2.54	12.10	87.90	0.172
CODA	3.94	12.49	87.51	0.162
HRS	55.19	18.83	81.17	(base)
War Babies	14.22	16.93	83.07	0.049
Early Baby Boomers	15.35	17.06	82.94	0.045
Mid Baby Boomers	8.76	16.32	83.68	0.064
Proxy help (%)				
Respondent answered	93.00	17.70	82.30	(base)
Respondent answered (got help writing)	5.94	16.39	83.61	0.034
Someone else answered	1.06	21.55	78.45	−0.101
Education level (%)				
Less than high school	13.38	21.18	78.82	−0.107
GED	4.75	19.84	80.16	−0.072
High school	31.46	17.14	82.86	(base)
Some college	24.43	18.12	81.88	−0.026
College and above	25.98	15.66	84.34	0.039
Missingness in the LHMS (%)				
Missing on less than 10% of questions	75.23	16.77	83.23	(base)
Missing on at least 10% of questions	24.77	20.38	79.62	0.097
Ability and motivation variables				
Cognitive score (mean, *SE*)	15.20 (4.20)	14.70 (4.20)	15.30 (4.20)	−0.133
Conscientiousness (mean, *SE*)	3.30 (0.40)	3.30 (0.40)	3.30 (0.40)	−0.083
Job history variables				
Number of jobs reported in HRS core (mean *SE*)	1.80 (1.40)	2.40 (1.50)	1.60 (1.40)	0.535
Length of recall (mean, *SE*)	10.50 (7.10)	11.90 (7.10)	10.20 (7.00)	0.236
Job schedule (%)				
Full-time	66.67	18.33	81.67	0.052
Part-time	33.33	16.32	83.68	(base)
Job tenure (%)				
<1 year	2.82	50.57	49.43	−0.160
≥1 and <5 years	7.60	42.65	57.34	(base)
≥5 years	39.31	19.91	80.09	0.460
Not working	50.27	10.28	89.72	0.655

*Note*: AHEAD = Assets and Health Dynamics of the Oldest Old; CODA = Children of the Depression; GED = General Educational Development; HRS = Health and Retirement Study; LHMS = Life History Mail Survey; *SE* = standard error.

### Measures

#### Dependent variables

*Work status match rate.—*As noted, in the LHMS, participants were asked if they had done paid work for 1 year or more since leaving full-time education. If yes, they were asked to complete a job grid that asked them to list the job title, information on employer or business type, start and end date, full-time or part-time status, and what they did after leaving the job for up to 10 jobs held. In the HRS core interview, respondents were asked if they are currently working and if yes, were asked for their job titles, information about employer and business type, typical hours per week, start year, and month for current job. We identified work status matches by comparing person-year observations from respondents who reported working in the LHMS in a particular year to work status in the HRS core for that same year. We created a binary variable for a match rate equal to one if both data sources indicated that the respondent was working in that year or if both sources indicated that the respondent was not working in that year, and zero otherwise.

**Figure 1.**
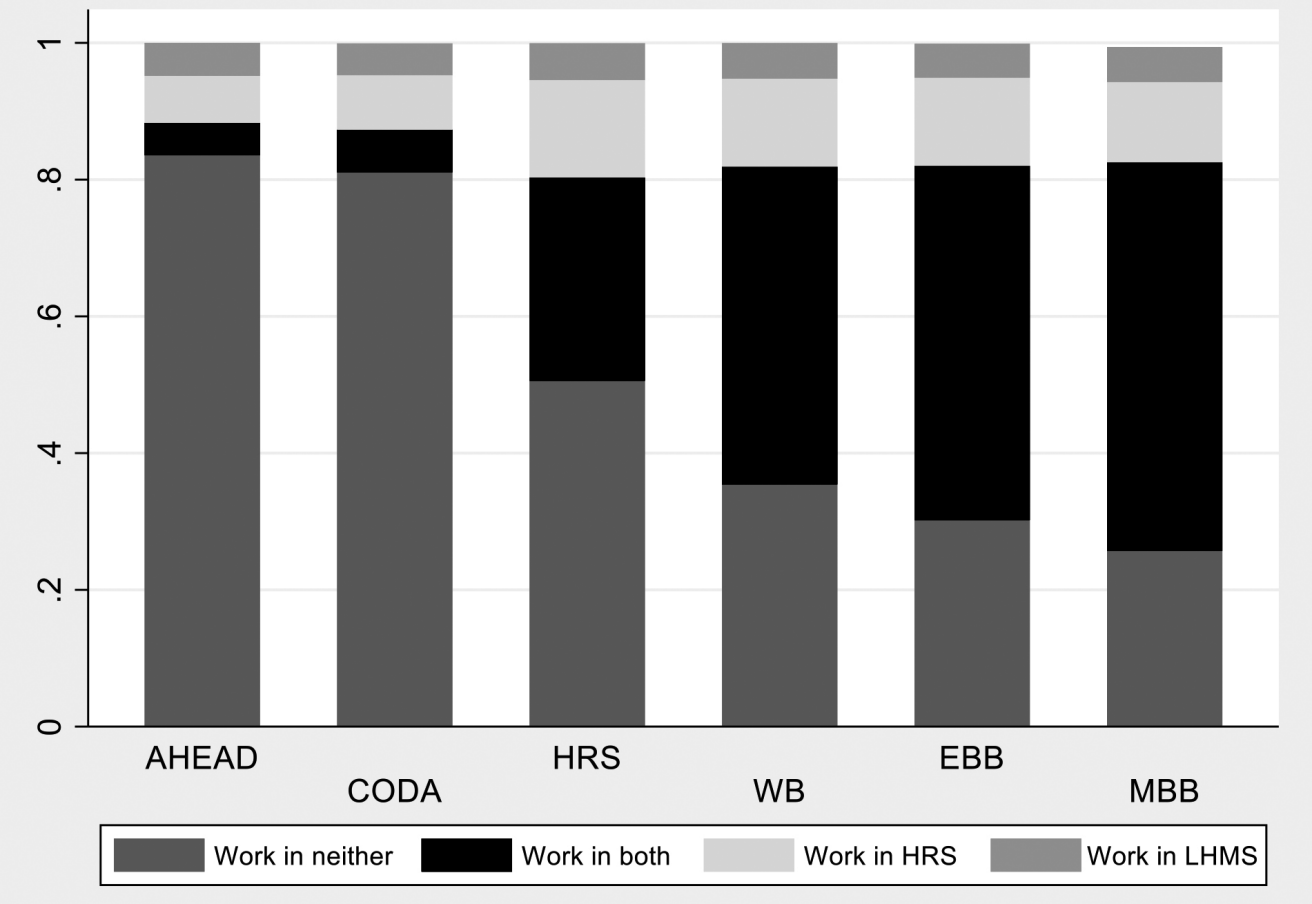
Person-year observations from respondents who were working in a particular LHMS year compared to HRS Core working status for that same year. EBB = early baby boomers; HRS = Health and Retirement Study; LHMS = Life History Mail Survey; MBB = mid baby boomers; WB = war babies. Late baby boomers were excluded due to low frequency.

For descriptive analyses of match rates (shown in Tables 2, 3A, and 3B, and Figure 1), we also considered the source of work information, whether from the LHMS or the HRS core, and created four variables indicating no work reported in both sources, work reported in both sources, work reported in the HRS core only, and work reported in the LHMS only. We also divided the sample into the following LHMS work history categories: (a) respondents with one or more jobs and no LHMS missing job start or end dates, (b) respondents with one or more jobs and some missing LHMS job start or end dates, (c) respondents who reported never working more than 1 year in the LHMS, and (d) respondents who reported working more than 1 year in the LHMS but provided no further job information.

**Table 2.** Work Status Match Rates, Overall and by LHMS Work History Category

Study variables	Overall match rate	Match rate by LHMS work history category			
		1+ jobs, complete date info.	1+ jobs, incomplete date info.	Never worked >1 year	No job info. provided
Work status by data source					
No work reported in both (%)	45.10	45.06	13.75	72.26	61.52
Work reported in both (%)	37.24	40.36	65.57	11.74	0.00
Work reported in HRS Core only (%)	12.49	9.57	8.55	13.35	38.48
Work reported in LHMS only (%)	5.17	5.01	12.14	2.65	0.00
Overall match rat*e*	82.34	85.42	79.32	84.00	61.52
Observations (person-year)	74,751	53,542	8,495	5,580	7,134
Observations (# of individuals)	8,648	6,027	1,196	630	795

*Note*: HRS = Health and Retirement Study; LHMS = Life History Mail Survey.

**Table 3.** Match Rates by Tenure (Incomplete + Complete LHMS Job Data)

Match status	Tenure based on HRS core		
	Less than 1 year	1–5 years	5 years or more
Work reported in both (%)	57.56	65.19	87.08
Work reported in HRS Core only (%)	42.44	34.81	12.92
Observations (person-year)	1,753	4,861	26,414
Observations (# of individuals)	249	673	4,633

*Notes*: HRS = Health and Retirement Study; LHMS = Life History Mail Survey.

This table includes person-year observations in which (1) the HRS Core data indicate that the respondent was working, and (2) the LHMS job grid contained information for at least one job, including at least job one start or end date. This sample includes respondents with both complete and incomplete LHMS job data.

*Occupation and industry match rate.—*The main focus of this manuscript is working status match rates; however, it was also possible to compare occupation and industry in a limited date range. Therefore, we conducted a set of analyses with the available data to provide a sense of how well these reports line up. HRS uses information on job titles and industries to assign Census industry and occupation codes. Information on the coding of occupation and industry is available in user guides for the LHMS (Smith et al., 2023a) and the HRS core (Nolte et al., 2016). We identified occupation and industry matches by comparing observations at the person-year level from respondents who reported their occupations and industries in the LHMS in a particular year to the occupations and industries reported in the HRS core for that same year. For the occupation matches, we created a binary variable equal to one if both data sources indicated the same two-digit occupation code and zero otherwise. Similarly, we also created a binary variable for industry matches based on the two-digit industry codes. However, we could only calculate occupation and industry match rates for a restricted date range given changes over time in the HRS coding of occupation and industry. Specifically, all of the jobs reported in the LHMS have been coded using the 2010 Census Occupation and 2007 Census Industry codes. Jobs reported in the HRS core from 1992 to 2008 were coded using earlier versions of the Census Occupation and Industry coding schemes that would not be comparable. From 2010 onward, HRS has coded occupation and industry using the 2010 Census Occupation and 2007 Census Industry codes. Thus, we could match information on industry and occupation in the core to the LHMS for the time frame 2010–2016. In addition, a related project allowed us to recode all of the 1992 coded data to the 2010 Census Occupation and 2007 Census Industry codes, which we compared separately.

#### Independent variables

Job history variables included number of reported jobs, recall length, job schedule, and job tenure. Number of jobs reported in the HRS core after enrollment in the biennial survey was a continuous variable that ranged from 0 to 11. This was a constructed variable based on respondents’ answers to whether they were working in a given year and whether they were working with the same employer as the previous wave. Recall length was a continuous variable ranging from 1 to 25 years for the length of time between job(s) reported in the HRS core and in the LHMS. We also included an indicator for the work schedule for each job coded as full-time = 1 and part-time = 0. Job tenure for each job held after enrollment was a constructed variable based on whether respondents reported working with the same employer as the last wave and their current job tenure. To evaluate potential nonlinearity in the connection between job tenure and match rate, and because the LHMS asked respondents to only report on jobs that lasted longer than 1 year, we converted this into a categorical variable coded 0 if tenure was less than 1 year, 1 if tenure was greater than or equal to 1 year and less than 5 years, and 2 if tenure was greater than or equal to 5 years, and 3 for not working.

Personal characteristic variables were cognitive function and conscientiousness. Cognitive functioning was a continuous variable ranging from 0 to 27 with a higher score indicating a higher level of cognitive functioning. Conscientiousness was a 10-item subscale of the “Big 5” Personality inventory based on MIDUS (Lachman & Weaver, 1997) and the International Personality Item Pool (http://ipip.ori.org; Smith et al., 2023b). Respondents were asked how much each adjective described them: 1 = a lot, 2 = some, 3 = a little, 4 = not at all. The summary score was the average of the 10 items, reverse-coded so that a higher score indicated a higher level of conscientiousness. Because conscientiousness was evaluated on a random rotating half of the core sample every wave, we selected the most recent values available for each respondent as far back as 2012.

#### Controls

We included several controls drawn from the RAND HRS Longitudinal File. Age in 2017 was a continuous variable ranging from 58 to 108 years. Gender was coded as 0 = male, 1 = female. Race and ethnicity were a categorical variable coded 1 = Neither Black nor Hispanic, 2 = Hispanic, 3 = Non-Hispanic Black, and 4 = Non-Hispanic Other. Cohort was a categorical variable coded 1 = AHEAD, 2 = CODA, 3 = HRS, 4 = War Babies, 5 = Early Baby Boomers, 6 = Mid Baby Boomers. Proxy help was coded as a categorical variable with 1 = respondent answered, 2 = respondent answered, got help writing, and 3 = someone else answered. Educational level was a categorical variable coded as 1 = less than high school, 2 = GED, 3 = high school, 4 = some college, and 5 = college and above. Finally, we accounted for the overall level of item missingness of information reported in the LHMS, coded as 0 = missing less than 10% of other LHMS questions and 1 = missing at least 10% of other LHMS questions.

### Analysis

We first conducted descriptive analyses of all study variables in the full sample and by work status match rate (matching or not matching; Table 1). For rows reported in the format (mean *SE*), Cohen’s D is calculated as ([the mean of the variable for matched—the mean of the variable for not matched]/[*SE* of the variable for not matched]); otherwise, Cohen’s D is calculated as ([the mean of the match for the specific category—mean of match for base category]/[*SE* of match for base category]). We also examined the distribution of the cohort by the four categories of work status match rate (Figure 1). We next examined the overall match rate and the match rate by LHMS work history categories (Table 2). We examined work status match rates by job tenure (Table 3) and work schedule (Table 4).

**Table 4.** Match Rates by Full-Time/Part-Time Status

Match status	Schedule based on HRS core data	
	Full-time	Part-time
Work reported in both (%)	90.92	79.82
Work reported in HRS Core only (%)	9.08	20.18
Observations (person-year)	22,404	4,431
Observations (# of individuals)	4,477	807
Full-time reported in LHMS (%)	95.41	59.93
Part-time reported in LHMS (%)	4.59	40.07
Observations (person-year)	16,663	2,763
Observations (# of individuals)	3,496	527

*Notes*: HRS = Health and Retirement Study; LHMS = Life History Mail Survey.

The top 4 lines of this table includes person-year observations from respondents (1) who were working full-time or part-time according to the HRS Core data, and (2) for whom the LHMS job grid contained information for at least one job and included at least one start or end date. The bottom 4 lines includes person-year observations from respondents (1) who were working full-time or part-time according to the HRS Core data, and (2) for whom the LHMS job grid data indicated that the respondent was working at the time and provided a full-time versus part-time indicator for that job. This sample includes respondents with both complete and incomplete LHMS job data.

We conducted multivariate mixed effects logistic regressions of the binary indicator of work status match on a vector of variables related to our hypotheses (Table 5). We included a random intercept at the individual level, such that the probability of matching between the LHMS and the Core was allowed to vary across individuals, controlling for covariates. This model considers both within-person match status (i.e., does the same person remember certain jobs less or more well) and between-person differences such as age, education, etc. This specification is optimal given that we are comparing match rates at multiple time points per person. This modeling accounts for unmeasured individual-specific factors that may affect recall. These unmeasured factors would give rise to a correlation in the errors between observations within each person over time in a standard logistic regression model.

**Table 5.** Odds Ratios and Standard Errors from Mixed Effects Logistic Regression of Match Status

Study variable	Model 1	Model 2	Model 3	Model 4
	OR (*SE*)	OR (*SE*)	OR (*SE*)	OR (*SE*)
Age in 2017	0.99 (0.01)	0.98 (0.01)*	1.00 (0.01)	0.98 (0.01)
Gender				
Male	1.00 (—)	1.00 (—)	1.00 (—)	1.00 (—)
Female	1.21(0.07)***	1.37(0.08)***	1.17(0.07)**	1.30(0.08)***
Race/ethnicity				
Non-Hispanic White	1.00 (—)	1.00 (—)	1.00 (—)	1.00 (—)
Hispanic	0.61(0.06)***	0.57(0.06)***	0.63(0.06)***	0.60(0.06)***
Non-Hispanic Black	0.67(0.05)***	0.55(0.05)***	0.71(0.06)***	0.60(0.05)***
Non-Hispanic other	0.71(0.12)*	0.66(0.12)*	0.73(0.13)	0.70(0.13)
Cohort				
AHEAD	3.61(1.36)***	2.73(0.98)**	3.59(1.36)**	2.71(0.96)**
CODA	2.28 (0.41)***	1.36 (0.24)	2.30 (0.41)***	1.38 (0.24)
HRS	1.00 (—)	1.00 (—)	1.00 (—)	1.00 (—)
War babies	0.99 (0.11)	0.83 (0.10)	1.00 (0.11)	0.83 (0.10)
Early baby boomers	1.21 (0.21)	0.59 (0.10)**	1.25 (0.21)	0.61 (0.11)**
Mid baby boomers	1.43 (0.34)	0.53 (0.13)**	1.51 (0.35)	0.57 (0.14)*
Proxy help				
Respondent answered	1.00 (—)	1.00 (—)	1.00 (—)	1.00 (—)
Respondent answered (got help writing)	1.29 (0.16)*	1.02 (0.13)	1.40 (0.17)**	1.15 (0.14)
Someone else answered	0.63(0.17)	0.57(0.15)*	0.70(0.19)	0.67(0.17)
Education level				
Less than high-school	0.83(0.08)*	0.78(0.08)**	0.89(0.09)	0.87(0.09)
GED	0.75(0.10)*	0.79(0.10)	0.78(0.10)*	0.83(0.11)
High-school graduate	1.00 (—)	1.00 (—)	1.00 (—)	1.00 (—)
Some college	0.90 (0.06)	0.94 (0.07)	0.87 (0.06)*	0.90 (0.07)
College and above	1.14 (0.08)	1.27 (0.09)***	1.05 (0.08)	1.14 (0.09)
Missingness				
Missing < 10% = 0	1.00 (—)	1.00 (—)	1.00 (—)	1.00 (—)
Missing at least 10% = 1	0.80 (0.05)***	0.72(0.05)***	0.83 (0.06)***	0.75 (0.05)***
Cognitive score			1.03 (0.01)***	1.04 (0.01)***
Conscientiousness			1.18 (0.08)*	1.23 (0.09)**
Number of jobs		0.64 (0.01)***		0.64 (0.01)***
Length of recall		0.95 (0.00)***		0.95 (0.00)***
Schedule				
Part-time		1.00 (—)		1.00 (—)
Full-time		6.68 (0.50)***		6.66 (0.50)***
Job tenure				
<1 year		0.71 (0.06)***		0.71 (0.06)***
≥1 year and <5 years		1.00 (—)		1.00 (—)
≥5 years		2.64 (0.21)***		2.62 (0.21)***
Not working		14.74 (1.50)***		14.78 (1.50)***
Lnsig2u	3.91 (0.13)***	3.80 (0.14)***	3.88 (0.13)***	3.75 (0.14)***
Observations	74,751	74,751	74,751	74,751
Individuals	8,648	8,648	8,648	8,648
Log likelihood	−28,706.369	−25,299.197	−28,695.303	−25,277.819
Rho	0.543	0.536	0.541	0.533

*Notes*: AHEAD = Assets and Health Dynamics of the Oldest Old; CODA = Children of the Depression; GED = General Educational Development; HRS = Health and Retirement Study; LHMS = Life History Mail Survey; *SE* = standard error.

*p < .05, **p < .01, ***p < .001.

Last, we conducted a limited set of analyses to provide suggestive evidence on the match of occupation and industry for the 1992 wave and for the time frame 2010 through 2016 (available in Supplementary Tables 3 and 4). All analyses were conducted in Stata 17.0.

### Sensitivity Analyses

We conducted sensitivity analyses that excluded participants with incomplete LHMS job history data to check the robustness of our results with regard to the bivariate associations with work schedule and job tenure. The restricted sample was 6,027 individuals with 53,542 person-years. These results are shown in the Supplementary Tables 1 and 2. Note that the overall work status match rate for this restricted sample appears in Table 2 of the main paper.

## Results

### Sample Characteristics and Match Rate Distributions

Table 1 shows descriptive statistics for all study variables both for the full sample and by match status (not matching and matching). The overall mean age (in 2017) was 74.70 years. About 61.79% of the respondents were female, 82.12% were White, 8.57% were Hispanic, and a quarter of respondents graduated from college. The average cognitive score was 15.20 (4.20), which corresponds to the normal range. Conscientiousness was 3.30 (0.40) on average, corresponding to “some” to “a lot.” The HRS cohort represented the majority of the sample (55.19%), followed by War Babies (14.22%), Early Baby Boomers (15.35%), and Mid Baby Boomers (8.76%). About 98.94% of the LHMS surveys were answered by the respondents themselves, and less than a quarter of all respondents left more than 10.00% of the questions unanswered in the LHMS.

Turning to the second and third columns in Table 1, women had higher match rates compared to men (83.08% vs 81.14%, *p* < .0001). Note that the sample used to conduct comparisons is the person-year (long) format; therefore, it is important to consider the size of the differences in means rather than focus exclusively on statistical significance (which is necessarily boosted owing to the large sample). We report Cohen’s D in the table to give the reader a sense of the magnitude of the mean differences. Effect sizes that are less than 0.2 are considered “very small,” “small” in the range of 0.2 to less than 0.5, “medium” in the range of greater or equal to 0.5 and less than 0.8. Coefficients from 0.8 to 1.00 are considered “large” or “very large.” Whites had higher match rates than Blacks (82.93% vs 79.17%, *p* < .0001), and non-Hispanics had higher match rates compared to Hispanics (82.66% vs 78.83%, *p* < .0001). Respondents with college and higher levels of education had higher match rates than respondents with high school education (84.34% vs 82.86%, *p* < .0001). The mean cognitive score was higher in the matching group (15.30 vs 14.70, *p* < .0001). Not surprisingly, there was a larger fraction of matches in the group with less than 10.00% missing on other sections of the LHMS questionnaire (83.23 vs 79.62, *p* < .0001).

Respondents who reported working for 1–5 years or more than 5 years had higher match rates compared to respondents who reported working for less than a year (57.34% and 80.09% vs 49.43%, respectively*).* Likewise, there was a higher mean number of jobs in the group not matching compared to matching (2.40 vs 1.60, *p* < .0001). The mean length of recall was also associated with match status with a higher length of recall in the not-matching group compared to matching (11.90 vs 10.20 years, *p* < .0001). Note that despite their statistical significance, the effect sizes (see column 4 in Table 1) are mostly in the range of less than 0.2, suggestive of very small effects.

Table 2 shows the work status match rates, both overall and by LHMS work history category. The overall work status match rate was 82.34%. Of the nonmatches, 70.73% reported working in the HRS core but not in the LHMS, while 29.27% reported working in LHMS and not in the HRS core. Respondents who provided complete LHMS job history data had higher match rates compared to respondents with incomplete or missing job history data (85.42% vs 79.32% and 61.52%, respectively). The match rate reported by those who did not work for more than a year in LHMS was similar to the match rate for respondents with a complete job history (84.00% vs 85.42%); however, more people reported working in the HRS core, and not in the LHMS, which is reasonable given that the LHMS only asked about jobs that had been held for more than 1 year.

Figure 1 shows the distribution of the cohort by the four categories of work status match rate. Not surprisingly, the AHEAD and CODA cohorts, with very high proportions of “worked in neither” relative to other cohorts, had higher match rates compared to the other cohorts.

Table 3 shows the work status match rates by job tenure based on the core. As expected, match rates increased with job tenure (87.08% for jobs held 5 years and more). Table 4 shows the works status match rate by work schedule. Similarly, as expected, full-time jobs had higher match rates compared to other schedules (90.92% vs 79.82%). About 95.41% of respondents who reported working full-time jobs in the core also reported working full-time in the LHMS, while only 40.07% of respondents who reported working other than full-time in the core also reported working part-time jobs in LHMS.

### Multivariate Analysis of Work Status Match Rates

Table 5 shows the results of the mixed effects logistic regression evaluating four models.

Model 1 (first column) shows the relationship between the baseline set of controls and the work status match rate. Interestingly, age was not associated with the match rate. Women were associated with an increased probability of a match (OR: 1.21, *p* < .001) relative to men. Respondents of Hispanic ethnicity (OR: 0.61, *p* < .001) and non-Hispanic Black (OR: 0.67, *p* < .001) respondents had lower match rates than non-Hispanic White respondents. Less than high school education and GED were associated with lower match rates relative to high school graduation (less than high school OR: 0.83, *p* < .05; GED OR: 0.75, *p* < .05). Compared to the HRS cohort, members of the AHEAD (OR: 3.61, *p* < .001) and CODA (OR: 2.28, *p* < .001) cohorts had better match rates, in line with the bivariate associations shown in Figure 1. Respondents who got help writing in their answers on the LHMS had higher match rates than those who answered completely on their own (OR: 1.29, *p* < .05). Last, respondents whose LHMS questionnaires contained at least 10.00% missing item-level responses to closed-ended items that were asked of all respondents had lower match rates (OR: 0.80, *p* < .001). Rho, or the proportion of total variance explained by the individual-level variance, is 0.54, indicating that there is a significant amount of unexplained variation that is attributable to unmeasured individual-level factors.

Model 2 (Table 5, column 2) added variables related to features of the respondent’s job history. We found support for hypothesis *H1a*, that a larger number of jobs was associated with a lower job status match rate (OR: 0.64, *p* < .001). Providing support for hypothesis *H1b*, we found an inverse association between the duration of time since the job was reported in the HRS and the 2017 LHMS and match rate (OR: 0.95, *p* < .001). In line with *H1c*, jobs reported as full-time according to HRS data were more likely to match the LHMS reports compared to part-time (OR: 6.68, *p* < .001). Last, providing support for hypothesis *H1d*, jobs held for less than 1 year according to HRS core data were less likely to match the LHMS reports (OR: 0.71, *p* < .001), and work for more than 5 years and non-working periods were much more likely to match the LHMS reports (OR 2.64, *p* < .001; OR: 14.74, *p* < .001). Adding these variables attenuated some of the effects of the cohort indicators relative to Model 1 (AHEAD OR 2.73, *p* < .01). The indicators on the Early Baby boomers and Mid Baby boomers indicated a decreased match rate, relative to the HRS cohort (OR 0.59, *p* < .01; OR 0.53, *p* < .01), suggesting that cohort effects captured in Model 1 were driven in part by variations in employment history HRS captured at each cohort’s baseline enrollment. College or greater education levels emerged as associated with higher match rates in model 2 (OR: 1.27*, p* < .001), relative to high school graduates. The variance explained between Models 1 and 2 changed very little.

Model 3 (Table 5, column 3) shows the association of conscientiousness and cognition and match rate, net of controls. Supporting hypothesis *H2a*, we found that a higher cognitive score was associated with a higher match rate (OR: 1.03, *p* < .001). Specifically, a one-point increase in cognition score was associated with a 3% increased probability of a match. Likewise, conscientiousness was positively associated with match (OR: 1.18, *p* < .05). College education or greater weakened compared to Model 1 and was no longer significant (OR: 1.05, *p* = .47), relative to high school graduates, suggesting that cognition and conscientiousness explained some of the education group-level differences found in Model 1. The patterns of results in the full model (Table 5, column 4) were substantively similar.

In sensitivity analyses that restricted the sample to respondents who provided complete job history data, we found similar match rates for jobs held 5 years and more as in the full sample (86.37% vs 80.09%; Supplementary Table 1). Likewise, full-time jobs had higher match rates compared to part-time jobs (91.04% vs 78.81%; Supplementary Table 2). The overall match rate for this restricted sample was 85.42% (Table 2).

We also evaluated occupation and industry match rates by tenure and full-time/part-time work status and found a 76.93% match rate for the industry category in LHMS and HRS core 2010–2016, and a 68.66% match rate for occupation in the same period (Supplementary Table 3). As with work status match rates, occupation and industry match rates increased with longer job tenure. The industry match rate by job tenure was higher than the occupation match rate (80.16% vs 70.07% for jobs held 5 years and more). The industry match rate between LHMS and HRS core 2010–2016 by full-time was similar to percentage of cases that by part-time jobs (77.70% vs 77.97%), while the occupation match rate for part-time was slightly higher than the occupation match rate for full-time jobs (69.22% vs 68.23%; Supplementary Table 3). The industry match rate for the 1992 wave with the LHMS was 77.64% and the occupation match rate was 68.68% (Supplementary Table 4), and similar patterns were found in relation to job tenure and full-time/part-time schedule.

## Discussion

This study took advantage of a retrospective life-history survey embedded in a national prospective study to evaluate the accuracy of retrospectively obtained employment information. We found a high agreement rate (about 83%) for work status, that is, the percent of cases where the respondents’ reports that they were working (or not working) in the LHMS matched reports from the HRS core. We also found that work status match rates differed according to aspects of job history and respondent characteristics, largely in directions predicted by theory and/or prior research. Specifically, we found support for the recall decay hypothesis, that jobs held longer ago were more difficult to recall accurately. In line with the concept of salience in autobiographical memory, jobs held for longer durations and that were full-time rather than part-time were recalled with greater accuracy. More complex job histories that involved a larger number of jobs were also associated with a lower match rate. Finally, given the complexity and overall task difficulty of the LHMS employment grid, we hypothesized that the personal characteristics of conscientiousness and cognitive function would affect the accuracy of the retrospective information. Indeed, we found that higher levels of conscientiousness and cognitive functioning were associated with a higher match rate.

Although most of the research investigating the reliability of retrospectively reported work histories has focused on unemployment, our findings are in accord with the results of these studies related to recall length and complexity of work history, indicated in this context by number of unemployment spells, and duration of the period of unemployment. For example, Pina-Sánchez et al. (2014) compared a Swedish register of unemployment to retrospective work histories collected through a survey and found that discrepancy between the two sources of information was positively associated with increased recall time and the number of unemployment spells experienced. In total, 54% of respondents reported the correct number of unemployment spells when asked 12–15 months after the fact, while only 7.5% did so when the recall period was extended to 11 years.

Similarly, Mathiowetz and Duncan (1988) compared administrative files from an American manufacturing firm with retrospective surveys from the Panel Study of Income Dynamics and found that longer spells of unemployment were reported more correctly relative to shorter spells. Using two sources of retrospective work histories—the British Household Panel Study and the Family and Working Lives Survey—Dex and McCulloch (1998) found that recall of unemployment events at the annual level was more reliable than recall of events at the monthly level. We found only one study that examined employment spells rather than just unemployment. Using the same data from the British Household Panel Study as Dex and McCulloch (1998), Jacobs (2002) compared initial employment data gathered in the first wave of the study with retrospective life-history data collected in the second wave a year later. Agreement between the initial and retrospective employment reports was 96.1% for men and 92.7% for women. This level of agreement is considerably higher than our findings but is likely due to the fact that the recall length was only 1 year. We found that recall length was associated with accuracy.

We used a limited set of waves of data in the HRS core that had the same Census coding regime available in the LHMS to examine the occupation and industry match. Wärneryd et al. (1991) compared retrospective occupational data collected in the Swedish Survey of Living Conditions—a large-scale, interview-based study conducted from 1979 to 1981—with data from censuses conducted in 1960, 1970, 1975, and 1980. The Nordic Occupational Classification, which separates occupations into groups using a three-digit system that grows more specific with each additional digit, was used in both data sets to classify the reported occupations finding agreement rates between 30% and almost 70% depending on the level of specificity of the coding regime compared. We found match rates for occupations similar to the higher end of this range.

Our findings should be viewed in light of several limitations. First, we compared concurrent job information collected prospectively from the core to that collected retrospectively in the LHMS; this means that we could only compare information on jobs held after age 50. Specifically, while the LHMS collects information on jobs held during respondents’ 20s through 40s, there was no corollary information from the core survey interview for these years. The literature on “memory bump” suggests that the 20s and 30s are likely to be the best-recalled period of life. We see evidence of this in the LHMS (Larkina et al., 2019), offering some reassurance that the accuracy of job information recalled at earlier life stages is likely to be at least comparable to that at later ages. Second, the LHMS did not contain a question about whether respondents had consulted any records to confirm information about their employment history, but future research should consider that direct measurement would be preferable. We found only modest effects of the job and personal characteristics we evaluated in association with match rates; however, this can be viewed as a welcome result in that accuracy does not appear to depend systematically on a range of characteristics that may be subjects of substantive study.

Last, we were only able to compare information on occupation and industry for the waves that contained matching Census information. A project is currently underway in the HRS to recode all of the jobs reported on in the prospective core survey according to the 2010 Census coding scheme. Future research could conduct a more comprehensive comparison of occupation and industry when these data are available. Other directions for future research could include expanding the evaluation of validity in the LHMS to residential history, which would be similar to job history in that it would be reasonable to compare information collected concurrently in the core to that collected retrospectively in the LHMS.

We conclude that a self-administered, paper-and-pencil questionnaire attempting to measure decades-long histories of autobiographically important dimensions of life can provide acceptable levels of agreement in employment information. We found important associations with job history and personal characteristics that may help inform the development of future efforts to collect life history information in a similar format.

## Supplementary Material

igae021_suppl_Supplementary_Tables_S1-S4
