# Supplementary material for: Can Retrospective Reports Provide Accurate Job History Information? A Comparison With Concurrent Reports in a National Prospective Study of Older Adults
Source: Innov Aging. 2024 Feb 23;8(3):igae021. doi: 10.1093/geroni/igae021 (PMC10976911; doi:10.1093/geroni/igae021)
Supplement: igae021_suppl_Supplementary_Tables_S1-S4 [file igae021_suppl_supplementary_tables_s1-s4.docx]

***Innovation in Aging* Supplementary Material: Sonnega, Amanda; Al-Hinai, Maymona; Chen, Qize; Helppie-McFall, Brooke; Smith, Jacqui. Can Retrospective Reports Provide Accurate Job History Information? A Comparison with Concurrent Reports in a National Prospective Study of Older Adults.**

**Supplementary Table 1: Match rates by tenure (only if complete LHMS job data)**

| **Match status** | **Tenure based on HRS** | | |
| --- | --- | --- | --- |
|  | **Less than 1 year** | **1-5 years** | **5 years or more** |
| Work reported in both (%) | 53.92 | 61.65 | 86.37 |
| Work reported in HRS Core only (%) | 46.08 | 38.35 | 13.63 |
| Observations (person-year) | 1,478 | 4,042 | 21,212 |
| Observations (# of individuals) | 204 | 546 | 3,774 |

*Note*. HRS= Health and Retirement Study; LHMS=Life History Mail Survey; This table includes person-year observations in which (1) the RAND data indicate that the respondent was working, and (2) the LHMS job grid contains information for at least one job and includes start and end dates for all jobs listed.

**Supplementary Table 2: Match rates by full-time status (only if complete LHMS job data)**

| **Match status** | **Schedule based on HRS** | |
| --- | --- | --- |
|  | **Full-time** | **Part-time** |
| Work reported in both (%) | 91.04 | 78.81 |
| Work reported in HRS Core only (%) | 8.96 | 21.19 |
| Observations (person-year) | 17,816 | 3,591 |
| Observations (# of individuals) | 3,624 | 659 |
| Full-time reported in LHMS (%) | 95.55 | 59.53 |
| Part-time reported in LHMS (%) | 4.45 | 40.47 |
| Observations (person-year) | 15,188 | 2,555 |
| Observations (# of individuals) | 3,147 | 478 |

*Note*. HRS= Health and Retirement Study; LHMS=Life History Mail Survey; The top panel of this table includes person-year observations from respondents (1) who were working full-time or part-time according to the HRS Core data, and (2) for whom the LHMS job grid contained information that for at least one job and included start and end dates for all jobs listed. The bottom panel includes person-year observations from respondents (1) who were working full-time or part-time according to the HRS Core data, and (2) for whom the LHMS job grid data also indicated that the respondent was working at the time and provided a full-time versus part-time indicator for that job.

**Supplementary Table 3: Industry and occupation match rates in 2010-2016 by tenure and by full-time status (incomplete + complete LHMS job data)**

| **Match status** | **Overall** | **Tenure based on HRS core** | | | **Schedule based on HRS core** | |
| --- | --- | --- | --- | --- | --- | --- |
|  |  | **Less than 1 year** | **1-5 years** | **5 years or more** | **Full-time** | **Part-time** |
| Industry category matches (%) | 76.93 | 54.95 | 70.01 | 80.16 | 77.70 | 77.97 |
| Observations (person-year) | 5,015 | 273 | 917 | 3,825 | 3,551 | 631 |
| Observations (# of individuals) | 980 | 37 | 127 | 816 | 803 | 134 |
| Occupation category matches (%) | 68.66 | 55.64 | 66.26 | 70.07 | 68.23 | 69.22 |
| Observations (person-year) | 5,073 | 257 | 904 | 3,912 | 3,604 | 640 |
| Observations (# of individuals) | 949 | 37 | 127 | 785 | 777 | 131 |

*Note*. HRS= Health and Retirement Study; LHMS=Life History Mail Survey; The left panel of this table includes person-year observations from respondents (1) who were working in a particular year from 2010 onward and had occupation or industry information in both the LHMS and RAND HRS datasets, and (2) for whom the LHMS job grid contained information for at least one job. The middle panel includes person-year observations from respondents in the left panel, broken down by job tenure. The right panel includes person-year observations from respondents in the left panel, broken down by work schedule.

**Supplementary Table 4: Industry and occupation match rates in 1992 by tenure and by full-time status (incomplete + complete LHMS job data)**

| **Match status** | **Overall** | **Tenure based on HRS core** | | | **Schedule based on HRS core** | |
| --- | --- | --- | --- | --- | --- | --- |
|  |  | **Less than 1 year** | **1-5 years** | **5 years or more** | **Full-time** | **Part-time** |
| Industry category matches (%) | 77.64 | 56.38 | 67.69 | 82.77 | 78.45 | 74.29 |
| Observations (person-year) | 1,820 | 149 | 359 | 1,306 | 1,527 | 245 |
| Observations (# of individuals) | 1,820 | 149 | 359 | 1,306 | 1,527 | 245 |
| Occupation category matches (%) | 68.68 | 60.00 | 65.42 | 70.70 | 67.93 | 73.93 |
| Observations (person-year) | 1,737 | 145 | 347 | 1,239 | 1,456 | 234 |
| Observations (# of individuals) | 1,737 | 145 | 347 | 1,239 | 1,456 | 234 |

*Note*. HRS= Health and Retirement Study; LHMS=Life History Mail Survey; The left panel of this table includes person-year observations from respondents (1) who have 2010 coded occupation/industry data from the 1992 interviews and LHMS occupation/industry data for that same year, and (2) for whom the LHMS job grid contained information for at least one job. The middle panel includes person-year observations from respondents in the left panel, broken down by job tenure. The right panel includes person-year observations from respondents in the left panel, broken down by work schedule.
